# Three-dimensional spatiotemporal analysis for the assessment of retinal capillary perfusion using a clinical OCT system

**DOI:** 10.1038/s41598-025-20659-6

**Published:** 2025-10-22

**Authors:** Yudan Chen, Jun Song, Hoyoung Jung, Tiffany Tse, Valerie Mok, Jennifer Tsang, Zaid Mammo, Myeong Jin Ju

**Affiliations:** 1 School of Biomedical Engineering, University of British Columbia, Vancouver, V6T 1Z4 Canada; 2 Department of Ophthalmology and Visual Sciences, University of British Columbia, Vancouver, V6T 1Z4 Canada

**Keywords:** Optical coherence tomography, Optical coherence tomography angiography, Retinal capillary perfusion heterogeneity, Coefficient of variation, Three-dimensional imaging, Biomedical engineering

## Abstract

Growing evidence suggests that subtle changes in retinal microcirculation may precede structural damage in vision-threatening diseases. Among these, perfusion heterogeneity within the retinal capillary network has emerged as a promising biomarker for early detection and disease monitoring. Recent advances in optical coherence tomography (OCT) and OCT-based angiography (OCTA) have enabled high-resolution, three-dimensional imaging of retinal morphology and vasculature. However, commercial systems remain limited in their ability to accurately analyze retinal perfusion dynamics due to reliance on proprietary and undisclosed post-processing algorithms. This paper introduces an effective protocol for spatial and temporal analysis of capillary perfusion heterogeneity using unprocessed OCTA volume data acquired by a commercial retinal imaging system. The proposed method employs a novel analysis utilizing the depth-resolved pixel-wise coefficient of variation (CoV) to quantitatively estimate retinal capillary perfusion heterogeneity. Comparison between the proposed method and conventional CoV analysis emphasizes the reliability of the new approach, incorporating depth-dependent signals. By using unprocessed OCTA data, the proposed method can provide more accurate measurements of retinal perfusion heterogeneity. Furthermore, the image processing techniques developed in this study could serve as a foundation for future research in other retinal vascular disorders.

## Introduction

Retinal blood flow is a highly dynamic process that exhibits pronounced spatial and temporal heterogeneity. This heterogeneity originates from multiple factors, most notably local metabolic demands, significantly affecting blood flow and pressure^1^. To facilitate this, retinal blood vessels demonstrate blood flow autoregulation^2^ that ensures adequate ocular blood flow to meet local metabolic demands, while protecting the capillaries from endothelial damage in the event of high systemic arterial pressure. Retinal blood flow is also altered by vasomotion, the spontaneous oscillation of retinal arteriole diameter^3^. While some degree of perfusion heterogeneity is normal in healthy individuals, impaired retinal perfusion autoregulation is hypothesized to contribute to the pathogenesis of several ocular diseases, including retinal vein occlusion^4^, diabetic retinopathy^5^, and glaucoma^6^. Many technologies have been developed to investigate pathological vascular changes. Fluorescein angiography has long been considered as the gold-standard method for 2D visualization of the retinal circulation^7,8^, but is an invasive modality requiring intravenous dye injection, and incompletely visualizes the deeper retinal capillaries. Laser Doppler flowmetry^9^ and laser speckle flowgraphy^10^ are non-invasive imaging techniques that use scattered light to quantify blood flow, but both modalities have limited image resolution^11,12^ and ability to resolve volumetric flow dynamics^11^.

Optical coherence tomography (OCT) is a widely used, non-invasive imaging modality for generating high-resolution 3D images which is particularly important in retinal imaging with various functional extensions. In particular, OCT-based angiography (OCTA)^13^ visualizes retinal vascular morphology by extracting the motion contrast from the movement of red blood cells. In addition to vascular structure visualization, temporal perfusion can also be evaluated through the variable interscan time analysis (VISTA) algorithm, which estimates the blood flow velocity between pairs of repeated scans with different interscan times^14,15^. However, robust VISTA implementation typically requires advanced OCT systems with high imaging speeds or custom scanning protocols that allow for a large number of repeated B-scans at a single transverse location, with short interscan times ranging from 1 ms to 1.5 ms^15–18^. These capabilities are generally only available on research-grade or prototype systems and are not standard in most commercially available OCT platforms. Moreover, commercially available clinical devices often do not provide access to the raw repeated B-scan data (BM-scans) nor allow users to customize scanning protocols, both of which are essential for implementing VISTA reliably. Another method that has previously been applied to OCTA to assess temporal perfusion is calculating the coefficient of variation (CoV), defined as the standard deviation divided by the mean of a corresponding input vector^1^ of the pixel-wise variability of vascular signals across consecutive OCTA images. However, previous studies have applied this CoV calculation solely based on the auto-generated and contrast refined *en face* OCTA images from the commercialized OCT system (Protocol A)^1,19^, which have been shown to result in inaccurate representations of the true perfusion heterogeneity. To compensate for the misrepresentation originating from the effect of built-in image refinement protocols of commercialized OCT systems, the other approach utilizes maximum intensity projection (MIP) of the OCTA volume data acquired through bypassing the image processing algorithms (Protocol B). However, this method could also be biased since the CoV value is not guaranteed to be computed from the signals located at the same depth, which originates from the potential fluctuations in OCTA intensities along the depth direction. In response to these limitations, we propose a novel volumetric CoV computation algorithm (Protocol C) for measuring the spatiotemporal variabilities of retinal capillary perfusion, verified by repeatability in healthy control subjects. By working directly with OCTA volume data, with the integration of feature-based 3D image registration algorithms, this approach aims to eliminate biases introduced by conventional CoV analysis.

## Materials and methods

### Imaging protocol

Cross-sectional measurements of variability in blood flow distribution from randomly selected 8 healthy subjects based on the inclusion criteria, which were designed to precisely assess the perfusion heterogeneity without any bias. The exclusion criteria include the presence of cardiovascular health conditions, vasoactive medication, active nicotine product consumption, as well as caffeine intake within 24 hours prior to the imaging session. Participants underwent three imaging sessions on different dates with at least 24 hours apart. The imaging was conducted at the Eye Care Centre of Vancouver General Hospital using PlexElite 9000 swept-source OCTA (Carl Zeiss Meditec Inc., Dublin, CA). A total of ten volumes were acquired consecutively for each eye, using a raster scanning pattern covering an area of 3 mm $\times$ 3 mm centered at the fovea. All participants were informed of the purpose and implications of the measurement, and written informed consent forms were collected from each subject before participating in this study. This study was approved by the research ethics board at the University of British Columbia (H19-03635) and followed the tenets of the Declaration of Helsinki. A total of ten *en face* OCTA images from each subject were extracted from the built-in layer segmentation algorithm of the system, encompassing from the upper boundary of the inner limiting membrane (ILM) to the lower boundary of the outer plexiform layer (OPL).

### 3D registration and averaging

**Fig. 1:**
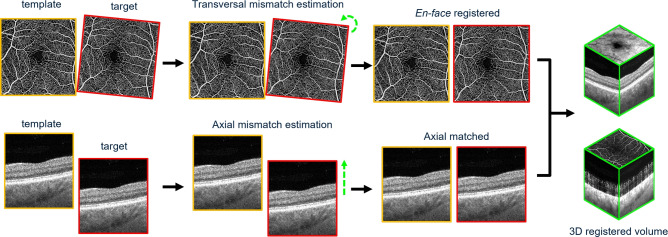
Illustration of multi-volume registration and averaging pipeline.

During imaging sessions, built-in motion correction (FastTrac, Carl Zeiss Meditec Inc., Dublin, CA) was activated to monitor the retinal movement and mitigate detected motion through automatic motion compensation. To maximize the overlapping field of view (FOV) across repeated scans, the built-in “Track to Prior” function was also enabled during image acquisition sessions, which automatically adjusts the parameters including chinrest position, fixation target, and scanning settings, to align with the prior scan and minimize the inter-scan variability. However, involuntary eye movements, physiological motion and poor fixation, could still be present, potentially misleading data interpretation in temporal variation analysis^20^. To address the residual inaccuracies, a custom-built 3D volume registration algorithm was applied to correct the spatial misalignments between serially acquired OCTA volumes that were not corrected by the automated motion compensation technique implemented in the system. Figure 1 demonstrates an overview of multi-volume 3D registration. Prior to the registration, the acquisition with the least motion artifact was selected as the template by evaluating the image quality on PlexElite-generated *en face* OCTA images. The transformation matrices from the template to the target images were obtained through both rigid and non-rigid registration algorithms. The rigid registration uses a hybrid invariant feature-based method that integrates Binary Robust Invariant Scalable Keypoints (BRISK)^21^ with Speeded Up Robust Feature (SURF)^22^, which are both widely used feature detector-descriptor schemes. This hybrid approach leverages the strengths of both algorithms to increase the transformation accuracy and robustness towards geometric deformation and location errors, as well as the inconsistencies in signal intensity and image contrast. Vasculature deformation between target and template was measured by diffeomorphic demons algorithm^23^ after rigid registration. The transformation matrices derived from this algorithm were simultaneously applied to the OCT and OCTA volumes along the axial direction. As the final step of 3D registration, a previously reported axial matching technique^24^ was applied to the volumes. This algorithm estimates the axial mismatches between the template and target OCT volumes, which demonstrates clear delineation of retinal layers compared to OCTA, to compensate for the discrepancies in the depth direction. To ensure consistent FOV after registration, the cropping boundaries were standardized to be 20 pixels on each side which corresponds to $\sim$ 200 $\upmu$m, accommodating the largest observed non-overlapping regions among all subjects. This standardized cropping strategy resulted in slightly reduced FOV but ensured comparability across subjects by maintaining identical final image dimensions.

### Volumetric coefficient of variation

CoV calculation methods used in this study for the estimation of retinal perfusion heterogeneity are summarized in Table 1, denoted as Protocols A to C.

**Table 1:** Summary of CoV calculation methods using different OCTA data representations and projection techniques.

Protocol	Data source	Projection type	Dimensions	Description
A	PlexElite-generated *en face* OCTA	Undisclosed	2D	CoV calculated from 2D *en face* image generated by a built-in algorithm
B	OCTA volume	MIP	2D	CoV calculated after applying MIP to the OCTA volume before built-in *en face* image generation algorithm
C	OCTA volume	Median intensity	3D	CoV calculated in 3D before applying a median projection

**Fig. 2:**
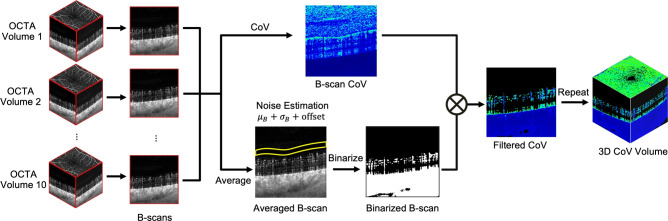
Illustration of the volumetric CoV computation pipeline using registered OCTA volumes.

Figure 2 demonstrates the volumetric CoV calculation steps, which estimates the perfusion variation between the B-scans from serially acquired OCTA volume data. The CoV was calculated using time-varying OCTA intensities ($I_k$) of the same pixel location defined by *x*, *y*, and *z* representing fast-scan, slow-scan, and depth locations respectively, across *N* ($N=10$) consecutive OCTA volumes as Eq. (1) states,

1 $$\begin{aligned} \textrm{CoV}(x,z;y) = \frac{\sigma (x,z;y)}{\bar{I}(x,z;y)} \end{aligned}$$

where $\textrm{CoV}(x,z;y)$ represents the value $\textrm{CoV}(x,z)$ calculated at the *y*-th B-scan, and $\bar{I}(x,z;y)$ and $\sigma (x,z;y)$ are mean and standard deviation of ten OCTA intensities at the identical voxel location defined by Eqs. (2) and (3):

2 $$\begin{aligned} \bar{I}(x,z;y) = \frac{1}{N}\sum _{k=1}^{N}I_k(x,z;y) \end{aligned}$$

3 $$\begin{aligned} \sigma (x,z;y) = \sqrt{\frac{1}{N-1}\sum _{k=1}^{N}\bigl [I_k(x,z;y)-\bar{I}(x,z;y)\bigr ]^{2}} \end{aligned}$$

To reduce the effects of noise signal, each B-scan CoV was multiplied by the binary mask generated by the averaged OCTA B-scan at the same *y*-th location. The noise floor was estimated from the background, *R*(*y*), spanning from ten to thirty pixels above the ILM, by the summation of the mean ($\bar{I}_{\textrm{noise}}(y)$) and standard deviation ($\sigma _{\textrm{noise}}(y)$) of pixel intensity, that are defined by Eqs. (4), (5).

4 $$\begin{aligned} \bar{I}_{\textrm{noise}}(y)\;=\;\frac{1}{|R(y)|}\sum _{(x,z)\in R(y)}\bar{I}(x,z;y) \end{aligned}$$

5 $$\begin{aligned} \qquad \sigma _{\textrm{noise}}(y)\;=\;\sqrt{\frac{1}{|R(y)|- 1}\sum _{(x,z)\in R(y)}\bigl [\bar{I}(x,z;y)-\bar{I}_{\textrm{noise}}(y)\bigr ]^2} \end{aligned}$$

Further elimination of noise signals was implemented by adding an empirically selected offset value $\delta$ to the binary threshold (*T*(*y*)) as stated in Eq. (6):

6 $$\begin{aligned} T(y)\;=\;\bar{I}_{\textrm{noise}}(y)\;+\;\sigma _{\textrm{noise}}(y)\;+\;\delta \end{aligned}$$

To select the offset value $\delta$, an initial value was first applied to generate a preliminary binary mask. This mask was multiplied by the averaged OCTA volume, producing a filtered OCTA volume. Subsequently, the filtered *en face* OCTA was extracted using segmentation of the nerve fiber layer (NFL) and OPL, and then qualitatively compared with the PlexElite-generated *en face* OCTA. The selection process involved incrementally increasing the value of $\delta$, where the final $\delta$ was determined as the highest possible value that effectively suppressed noise without compromising vascular integrity.

Based on the estimated *T*(*y*), a binary mask (*M*(*x*, *z*; *y*)) was derived as shown in Eq. (7):

7 $$\begin{aligned} M(x,z;y)\;=\; {\left\{ \begin{array}{ll} 1, & \bar{I}(x,z;y) > T(y),\\ 0, & \text {otherwise}, \end{array}\right. } \end{aligned}$$

The perfusion variation (*V*(*x*, *z*; *y*)), described by filtered CoV, was calculated by multiplying the B-scan CoV by the binary mask (Eq. (8)). Consecutive derivation of B-scan CoV was conducted for every *y* direction to expand into volumetric estimation of perfusion heterogeneity.

8 $$\begin{aligned} V(x,z;y)\;=\;\textrm{CoV}(x,z;y)\,\odot \,M(x,z;y). \end{aligned}$$

### Retinal layer and vessel segmentation

**Fig. 3:**
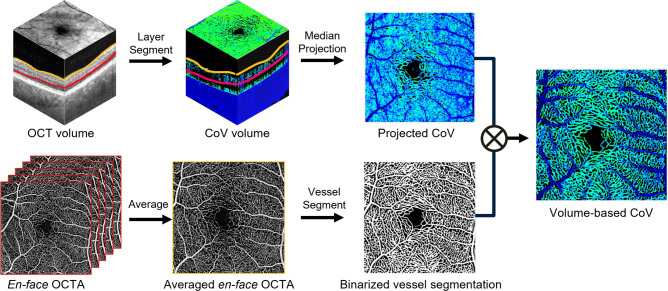
Illustration of the *en face* visualization pipeline.

For clear visualization of retinal perfusion heterogeneity, deep neural network (DNN)-based segmentation algorithms were implemented for *en face* image generation of volumetric CoV. The retinal layer segmentation was conducted from the averaged OCT volumes which ensures the precise and accurate delineation of depth-dependent retinal structures. This model detects the layer boundaries using OCT B-scan in a 2D basis, utilizing a novel LF-UNet architecture^25^, which is a hybrid model that combines the U-Net and a fully convolutional network (FCN), harnessing the strengths of both networks to enhance efficiency and robustness in detecting retinal layer boundaries. Based on the layer segmentation, morphological characteristics of the NFL and OPL were projected from the filtered CoV volume using median intensity projection.

Uncompensated residual noise was further suppressed by a DNN-based 2D vessel segmentation algorithm^26^. Ten PlexElite-generated *en face* OCTA images were averaged to further improve vascular contrast and vessel connectivity, which was fed into the neural network for generating a vascular probability map with the identical size. The probability maps were binarized with a threshold of 0.5, which corresponds to a confidence level of $50\%$ for identifying a pixel as a blood vessel. This method outperformed conventional vessel segmentation methods, such as simple thresholding or vesselness filters^26^, in their sensitivity to detect vessel boundaries. The projected image of CoV volume was multiplied by the binarized probability map to separate the vascular signals from static tissue and noise, resulting in enhanced visualization of the retinal vasculature. The capillary perfusion heterogeneity was manifested by the color-coded CoV values where red and blue indicate high and low variability, respectively.

### Code availability

Statistical analysis was performed using MATLAB R2023b (The MathWorks Inc., Natick, MA) with custom-built processing algorithms. A graphical user interface implementing the proposed pipeline is publicly available and can be accessed and installed from the GitHub repository CV3D_Perfusion.

## Results

### Comparison across different CoV measurements

For comprehensive validation of the proposed CoV measurement algorithm, data acquired from subjects were analyzed using three different protocols. The CoV maps for all eight subjects can be found in Supplementary Figure 1 available online.

**Fig. 4:**
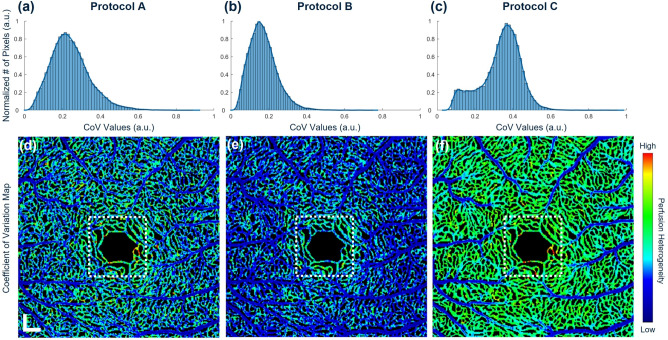
Histogram plots of the CoV values of a representative healthy subject computed with (**a**) Protocol A, (**b**) Protocol B and (**c**) Protocol C. Color-coded visualization of CoV using (**d**) Protocol A, (**e**) Protocol B and (**f**) Protocol C. Scale bar = 200 $\upmu \hbox {m}$.

Figure 4 shows representative perfusion heterogeneity of a 24-year-old healthy male using three different processing protocols. As shown in the figure, all methods demonstrate relatively high perfusion heterogeneities at the deep vascular plexus (DVP) while low at the superficial vascular plexus (SVP). Microvasculature with high variance appears as red near the foveal avascular zone (FAZ), but is only noticed from Protocols A and C, as shown in the white boxes in Figs. 4d and f. In addition, each method shows significantly different distributions of computed CoV values, where the Protocol C clearly distinguishes the main arterioles and venules from their smaller branches (Fig. 4f), while Protocols A and B exhibit scattered areas of moderate and high variance. Histogram plots of CoV values also demonstrate quantitative comparison among the protocols. In particular, the bimodal CoV distribution is observed from Fig. 4c while the others demonstrate a unimodal distribution.

**Fig. 5:**
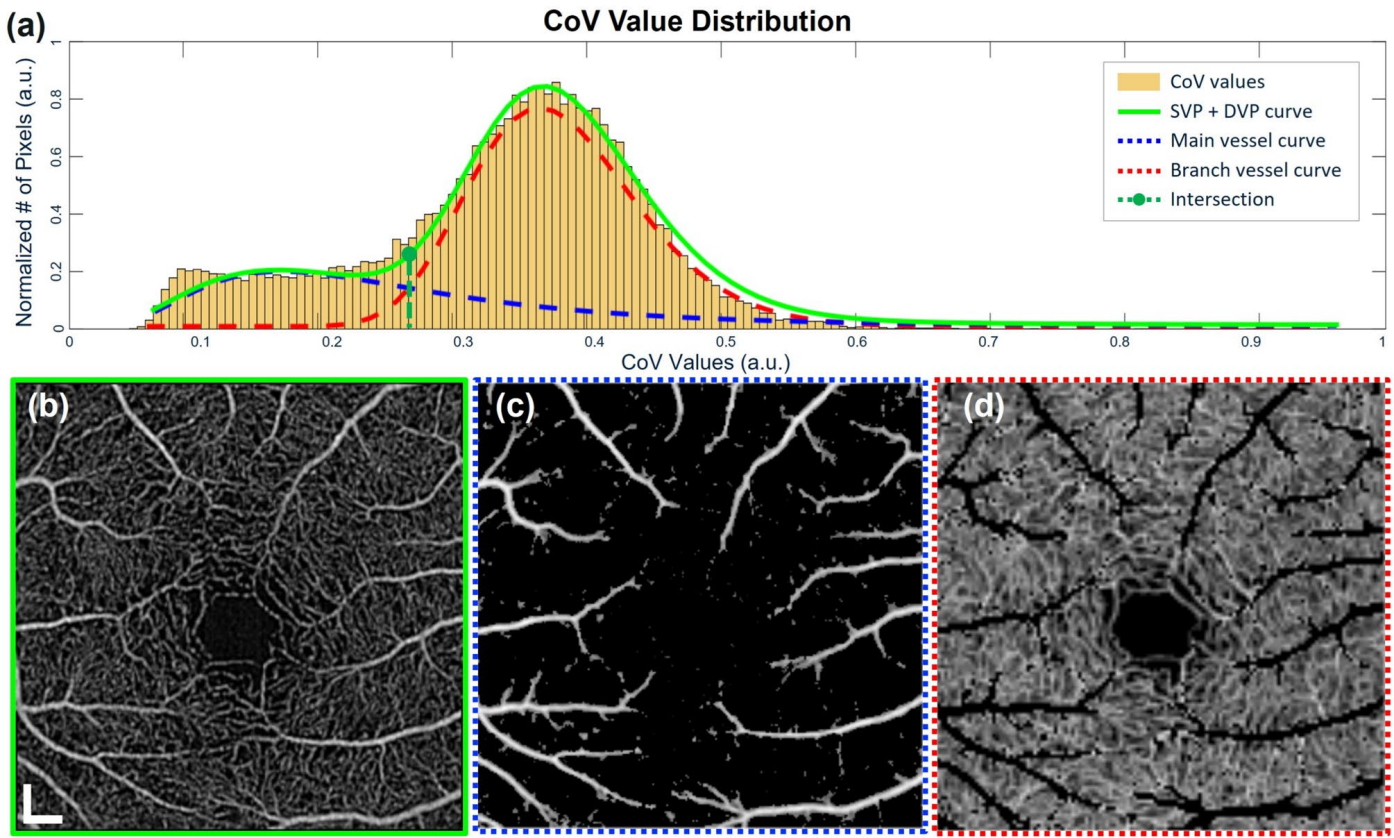
Illustration of vessel segmentation by using the intersection point derived from the CoV values calculated using Protocol C. (**a**) Histogram plot of the CoV values enveloped by a solid green line, which is the summation of the two estimated lognormal functions indicated by blue and red dotted line respectively. (**b**) *En face* OCTA spanning from SVP to DVP, (**c**) segmented arterioles and venules, (**d**) capillary branches of the arteriole and venule extracted based on the intersection point demonstrated in (**a**). Scale bar = 200 $\upmu \hbox {m}$.

Further analysis with volumetric CoV is presented in Fig. 5. Based on the assumption of a bimodal distribution, supported by two observed peaks in the histogram plot, the predicted model is defined by a weighted sum of two independent components, where each component is modeled by a lognormal probability density distribution, $P_{\text {LN}}(v)$, defined by Eq. (9):

9 $$\begin{aligned} P_{\text {LN}}(v) = \frac{1}{v\sigma \sqrt{2\pi }}\exp (-\frac{(\ln v - \mu )^2}{2\sigma ^2}) \end{aligned}$$

As shown in Eq. (10), the predict model, $P_{\text {pred}} (v_n)$, is therefore defined as a function of $v_n$, which corresponds to the CoV value at the center of the *n*-th histogram bin,

10 $$\begin{aligned} P_{\text {pred}} (v_n) = \lambda _1 \cdot \frac{1}{v_n\sigma _1 \sqrt{2\pi }}\exp \left( -\frac{(\ln v_n - \mu _1)^2}{2\sigma _1^2}\right) + \lambda _2 \cdot \frac{1}{v_n\sigma _2 \sqrt{2\pi }}\exp \left( -\frac{(\ln v_n -\mu _2)^2}{2\sigma _2^2}\right) \end{aligned}$$

where $\lambda _i$ is the weight, $\mu _i$ is the mean and $\sigma _i$ is the standard deviation of the two components. The initial value of the parameters was set to be:

$$\begin{aligned} \lambda _1 = \lambda _2 = 0.5 \end{aligned}$$

$$\begin{aligned} \sigma _1 = \sigma _2 = 0.5 \end{aligned}$$

$$\mu _1 = \ln {\overline{v_n}}, \quad \mu _2 = 1.5 \cdot \mu _1$$

where $\overline{v_n}$ is mean of $v_n$ across all histogram bins.

These parameters were iteratively optimized by solving a nonlinear curve-fitting problem using the MATLAB built-in function, lsqcurvefit (Eq. (11)), to minimize the least-squares difference between predicted model and the actual data:

11 $$\begin{aligned} \min _{\theta } \sum _{n=1}^N \left( P_{\text {pred}}(\theta ;\, v_n) - P_n \right) ^2 \end{aligned}$$

where $\theta$ is the set containing all parameters, including {$\lambda _1,\,\lambda _2,\,\mu _1,\,\mu _2,\,\sigma _1,\,\sigma _2$}, $P_{\text {pred}}(\theta ;\, v_n)$ is the predicted probability density at bin center $v_n$ with certain $\theta$ and $P_n$ is the actual value. Two estimated lognormal functions are illustrated by the red and blue dotted lines in Fig. 5a, with the green solid line representing their summation, effectively enveloping the histogram. Notably, the intersection of these two lognormal curves provides a statistical threshold that effectively separates the main and branching capillaries, reflecting inherent differences in their CoV values calculated by Protocol C. This threshold-based separation facilitates depth-dependent visualization of individual plexus as shown in Fig. 5b–d. This method enables clear differentiation and effective visualization of vessels at various axial locations.

### Repeatability and consistency

**Fig. 6:**
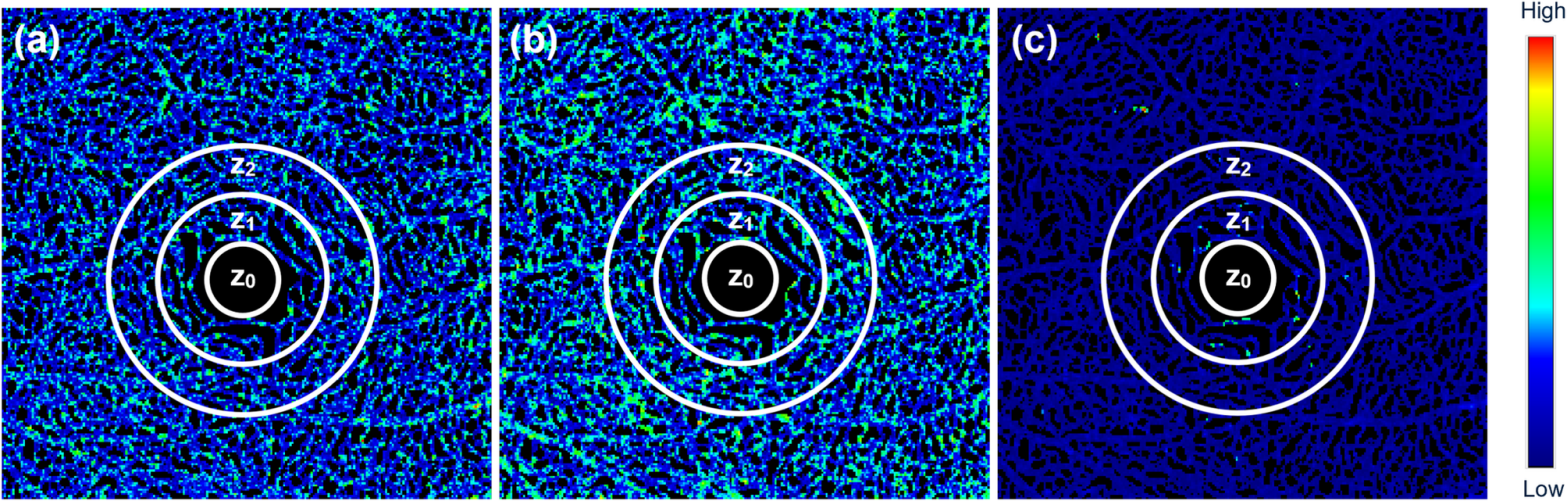
Illustration of variance of CoV across the time points computed with three approaches. (**a**) Protocol A, (**b**) Protocol B. (**c**) Protocol C. In all protocols, $z_0$, $z_1$, and $z_2$ represent the concentric areas covering the FAZ and with incremental distances of 0.5 mm and 1 mm to the boundary of $z_0$, respectively.

To evaluate the repeatability and consistency of the proposed method, the data acquisition and analysis were also conducted at three distinct time points to each subject. The resulting CoV metrics were registered to correct for spatial misalignment before comparison. Figure 6 shows the variations in CoV values across the time points, computed using the three different approaches. The variance was color-coded for clear visualization, with blue and red representing high and low consistencies, respectively. In Figs. 6a and b, results generated by Protocols A and B exhibit scattered areas of moderate and high inconsistencies over time reflecting potential misrepresentation of perfusion heterogeneity. In contrast, Protocol C yields predominantly blue regions (Fig. 6c), indicating a more reliable estimation of perfusion heterogeneity with less fluctuations in measurement over time. However, vessels near the FAZ show relatively higher fluctuations, as highlighted in green.

**Fig. 7:**
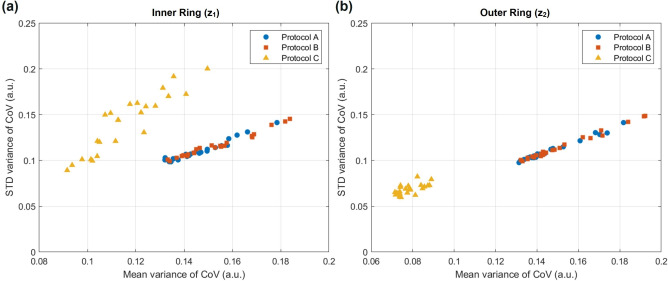
Scatter plots of mean and standard deviation (STD) of the variance of CoV values of (**a**) $z_1$ and (**b**) $z_2$ across the three time points for all subjects, with blue circles, red squares, and yellow triangles representing the CoV computed by Protocols A, B and C respectively.

For further validation of the proposed method, the variance-based approach was implemented for intersession repeatability and consistency assessment^27,28^. The variance between $V_1$, $V_2$ and $V_3$ from three separate imaging sessions was computed. For each subject, variance was determined for the three possible session pairs ($V_{1}$ $\rightarrow$ $V_{2}$, $V_{1}$ $\rightarrow$ $V_{3}$, and $V_{2}$ $\rightarrow$ $V_{3}$), yielding 24 variance estimates across eight subjects. To better investigate the spatially dependent characteristics of the perfusion heterogeneity as highlighted in Fig. 6, two rings centred at the fovea with a distance of 0.5 mm and 1 mm to the FAZ ($\text {z}_0$) were defined as $\text {z}_1$ and $\text {z}_2$ regions. The CoV values computed with Protocol C show low mean variances in both $\text {z}_1$ ($0.1158\pm 0.0150$) and $\text {z}_2$ ($0.0784\pm 0.0055$), compared to those obtained with Protocol A ($0.1466\pm 0.0116$ and $0.1464\pm 0.0138$, respectively) and Protocol B ($0.1526\pm 0.0146$ and $0.1520\pm 0.0178$, respectively), indicating high consistency. Within $\text {z}_1$ (Fig. 7a), the standard deviation of variance in CoV tends to be higher as a result of the rapid changes in perfusion heterogeneity of the microvasculature near the FAZ^19^.

On the other hand, the variance of CoV at $\text {z}_2$ (Fig. 7b) clearly distinguishes the proposed approach from the other methods since Protocol C consistently demonstrates low variance at $\text {z}_2$ while the other two protocols result in fluctuations as shown in Fig. 6.

## Discussion

The results of this study demonstrate the capability of reliable volumetric CoV analysis in characterizing the spatiotemporal dynamics of the retinal vascular plexuses. By leveraging the B-scan images providing cross-sectional views of the retina, our method maintains the depth information necessary for detailed capillary-specific analysis. This is particularly advantageous for assessing perfusion heterogeneity across different capillary networks, offering a comprehensive overview of variations in retinal blood flow.

**Fig. 8:**
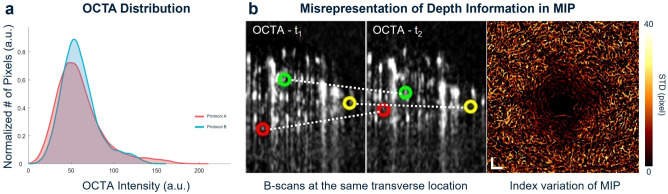
Limitations in previous methods. (**a**) Histogram plots of the OCTA intensity distribution of the data source for Protocol A (red) and Protocol B (blue). (**b**) Misrepresentation of depth information, visualized from repeated B-scans after registration and *en face* images. Each pair of circles annotated with the same color (red, green and yellow) connected by the white dashed line represents the inconsistent depth locations of maximum OCTA intensity at the identical A-scan. The *en face* image illustrated the variation in indices of the selected maximum value across repeated scans. Scale bar = 200 $\upmu \hbox {m}$.

Significant limitations of the previous approaches, Protocols A and B, are demonstrated in Fig. 8a. The PlexElite-generated *en face* OCTA images are known to have higher variabilities in pixel intensities originating from proprietary *en face* image rendering algorithms implemented in commercial OCT systems for enhancing vessel visibility. However, this intensity adjustment can contribute to the misrepresentation of time-varying OCTA signals in CoV computation, resulting in inaccurate estimations of perfusion heterogeneity. As shown in Fig. 8a, the pixel intensity distribution of the image used in Protocol A shows noticeable differences compared to the images extracted from the OCTA volume. In addition, Protocol B is prone to be affected by MIP artifacts that are caused by inconsistencies in the axial location of maximum OCTA intensity. As shown in Fig. 8b, the maximum values were inconsistently selected along the depth at the identical transverse location in the registered B-scans (OCTA-t$_1$ and OCTA-t$_2$). This inconsistency is more clearly visualized in the *en face* image of depth index variation, color-coded by its STD. Consequently, the CoV map generated using Protocol B often introduces sporadic distributions of high variations over the entire imaging FOV. This issue is particularly problematic around the FAZ, as even minor inconsistencies can significantly distort the appearance of high perfusion heterogeneities that are often indicative of pathological changes^19,29^. To address these limitations, Protocol C was developed to reduce system-induced biases by operating on minimally processed intensity data that are not affected by proprietary OCTA image processing. More importantly, Protocol C was intentionally designed to improve generalizability across different OCTA systems, regardless of variations in hardware configurations or vendor-specific post-processing algorithms by relying on a consistent computational framework and estimating the noise floor on a per-volume basis. While this study focuses on data from a single commercial system, future work will involve validating the generalizability of Protocol C across multiple OCTA systems from different manufacturers.

Volumetric CoV analysis can mitigate the bias of the other two protocols as shown in Fig. 4. The consistency and repeatability of the proposed protocol was evaluated by computing the variance of CoV calculated at different times. By definition, statistical variance reflects the degree of dispersion, indicating how much the CoV values fluctuate across repeated measurements. As shown in Fig. 6, lower variance in CoV across different time points reflects greater stability in the measurement. In this context, Protocol C demonstrates the most consistent CoV values over time, as evidenced by its lower variance compared to the other two protocols across $z_{1}$ and $z_{2}$.

The plots of STD and the mean variance ($\mu$) in Fig. 7 further reinforce the consistency of Protocol C. The data points at the outer ring ($z_{2}$) demonstrate a linear trend across the protocols, indicating that the variance of CoV calculated from Protocol C shows lower STD and $\mu$ compared to the other protocols. However, the inner ring ($z_{1}$) shows a slightly different result where the values are not linearly fit. This deviation possibly originates from the shunting vessels, which generally demonstrate high CoV values and are mostly located close to the FAZ. However, shunting vessels do not always exhibit high CoV values as the temporal variation of perfusion heterogeneity can fluctuate^30^. Based on this fact, the inner ring may show higher fluctuations in variance of CoV values that cause the STD and $\mu$ values calculated using Protocol C not to fit into a linear trend as the result of the outer ring does. The proposed method clearly visualizes the subtle changes in perfusion heterogeneity near the FAZ while presenting more consistent CoV values at other regions compared to Protocols A and B as demonstrated in Fig. 7. This reliability is based on the characteristics of the algorithm that extracts the calculated values by taking the depth information into account, which is not considered for the other two approaches. These findings highlight the better consistency of Protocol C while still capturing localized changes in areas with higher vascular variability. The CoV value distribution pattern with two peaks shown in Fig. 5a calculated by Protocol C indicates a clear distinction of vessels with different axial positions, enabling the classification of different capillary networks. This separation is particularly significant as perfusion heterogeneity in the form of shunting vessels is often observed near the FAZ among retinal vascular plexuses^19^.

Although the significance of volumetric CoV analysis and its efficacy over conventional perfusion heterogeneity measurement approaches have been presented, we acknowledge that this study has several limitations. First, the data used for this study was only acquired from healthy subjects whose findings do not include pathological conditions such as glaucoma and diabetic retinopathy, where vascular irregularities are more likely to be observed. In addition, the accuracy of the proposed method is highly dependent on the performance of the retinal layer and vessel segmentation. While the incorporation of a DNN-based method can improve efficiency and reliability, it requires a sufficiently large and diverse training dataset to improve robustness. In particular, data with weak contrast, pathology-induced abnormalities and high anatomical variability can be challenging for the current DNN model to effectively segment layers, in which cases manual correction may still be necessary to guarantee accuracy. Furthermore, this study focused on a relatively small scanning area centered on the fovea. Scaling the method to wider FOV could introduce additional challenges such as increased motion artifacts, which may affect CoV calculations. Currently, volumes with significant motion artifacts are manually identified and excluded to maintain the sufficient FOV for further analysis. However, future work will aim to develop automated motion detection and correction methods to improve robustness and facilitate analysis of wide-field OCTA datasets. For future studies, data will be collected from both healthy subjects and patients with retinal diseases to identify pathological characteristics in perfusion heterogeneity with larger cohorts.

## Supplementary Information

Supplementary Figure 1.

## Data Availability

The datasets generated during and/or analysed during the current study are not publicly available due to the ethical and privacy issues related to the human subjects but are available from the corresponding author on reasonable request.
