# Supplementary material for: Three-dimensional spatiotemporal analysis for the assessment of retinal capillary perfusion using a clinical OCT system
Source: Sci Rep. 2025 Oct 22;15:36849. doi: 10.1038/s41598-025-20659-6 (PMC12546696; doi:10.1038/s41598-025-20659-6)

Supplementary figures

Supplementary Figure 1: The CoV maps generated from all eight subjects using three different protocols.

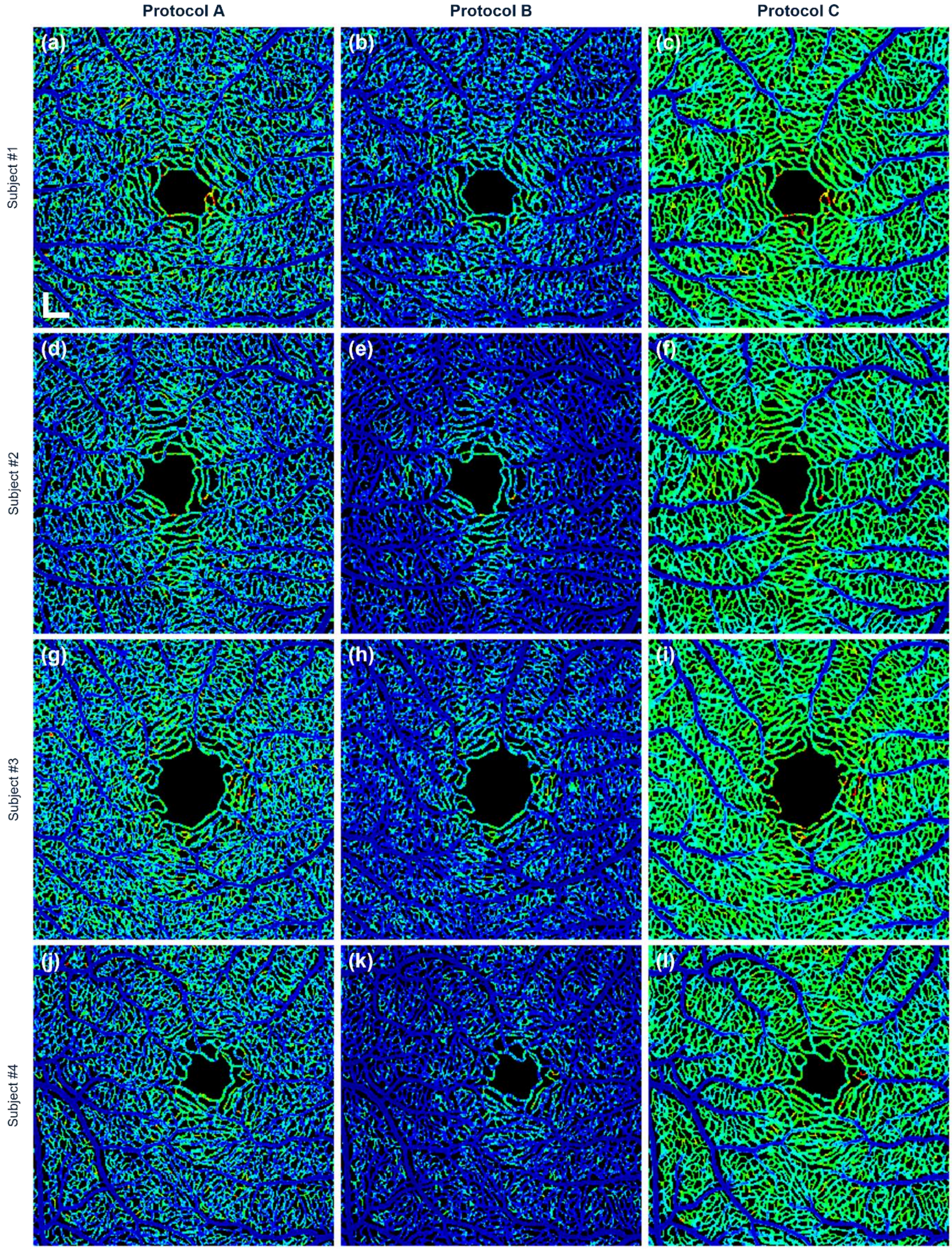

(Continued)

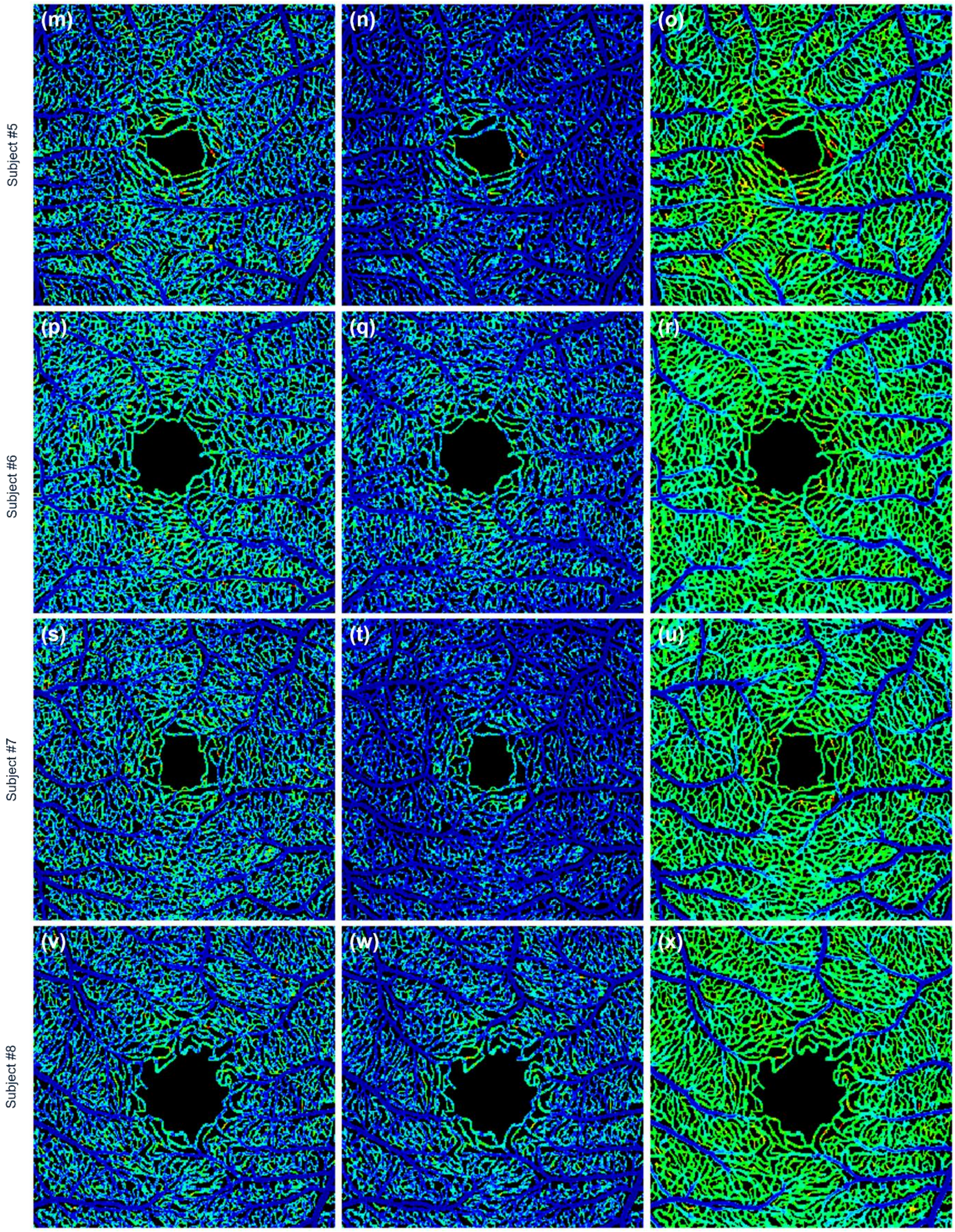

Supplement: Supplementary file 1 — Supplementary Figure 1. [file 41598_2025_20659_MOESM1_ESM.pdf]
